# SMN and coilin negatively regulate dyskerin association with telomerase RNA

**DOI:** 10.1242/bio.018804

**Published:** 2016-05-23

**Authors:** Aaron R. Poole, Michael D. Hebert

**Affiliations:** Department of Biochemistry, The University of Mississippi Medical Center, Jackson, MS 39216-4505, USA

**Keywords:** Cajal body, SMN, Coilin, Telomerase

## Abstract

Telomerase is a ribonucleoprotein comprising telomerase RNA and associated proteins. The formation of the telomerase holoenzyme takes place in the Cajal body (CB), a subnuclear domain that participates in the formation of ribonucleoproteins. CBs also contribute to the delivery of telomerase to telomeres. The protein WRAP53 is enriched within the CB and is instrumental for the targeting of telomerase RNA to CBs. Two other CB proteins, SMN and coilin, are also suspected of taking part in some aspect of telomerase biogenesis. Here we demonstrate newly discovered associations between SMN and coilin with telomerase components, and further show that reduction of SMN or coilin is correlated with increased association of telomerase RNA with one these components, dyskerin. These findings argue that SMN and coilin may negatively regulate the formation of telomerase. Furthermore, clinically defined SMN mutants found in individuals with spinal muscular atrophy are altered in their association with telomerase complex proteins. Additionally, we observe that a coilin derivative also associates with dyskerin, and the amount of this protein in the complex is regulated by SMN, WRAP53 and coilin levels. Collectively, our findings bolster the link between SMN, coilin and the coilin derivative in the biogenesis of telomerase.

## Abstract (precis)

**Summary:** Newly-discovered interactions between the Cajal body proteins SMN and coilin with the telomerase complex are reported. These findings suggest that SMN and coilin participate in early steps of telomerase biogenesis.

## INTRODUCTION

Telomerase biogenesis, like that for other ribonucleoproteins (RNPs), requires the ordered assembly of protein factors onto a non-coding RNA (Egan and Collins, 2012). The Cajal body (CB), a subnuclear domain, contributes to the assembly of telomerase and other RNPs, including small nuclear RNPs which are involved in pre-mRNA splicing, and small Cajal body-specific RNPs (scaRNPs) which modify snRNPs (Machyna et al., 2013). The RNA component of telomerase, known as TERC or TR (hTR if referring to that in human) is an H/ACA class of small non-coding RNA. H/ACA motifs are also found in small nucleolar RNAs (snoRNAs) and small Cajal body-specific RNAs (scaRNAs). In addition to the H/ACA motifs, hTR contains stem loops and other *cis* elements that recruit various protein components. One of these *cis* elements is the CAB box, which is bound by the protein WRAP53 (Tycowski et al., 2009; Venteicher et al., 2009). Since WRAP53 localizes to the CB, the interaction of WRAP53 with scaRNAs that contain a CAB box provides a mechanism for the recruitment of these RNAs to the CB. Like scaRNAs, hTR is enriched within the CB. Because of the similarities between hTR and box H/ACA scaRNAs, hTR is considered a scaRNA. Mature telomerase contains hTR in a complex with the core proteins Nop10, Nhp2, Gar1, and dyskerin along with the telomerase reverse transcriptase (hTERT) (Trahan and Dragon, 2009). The CB is thought to be the assembly point for the incorporation of hTERT into the nascent telomerase complex. In the early stages of telomerase formation, the complex contains NAF1, which is later replaced by Gar1 (Egan and Collins, 2012). Hence hTR associated with NAF1 or Gar1 is a marker for the premature or mature complex, respectively. Disruptions in the formation of telomerase result in the disease dyskeratosis congenita.

Previous work has implicated two other CB-enriched proteins, SMN and coilin, in telomerase biogenesis. SMN, the survivor of motor neuron protein, has been extensively characterized in terms of its contribution to small nuclear RNP (snRNP) formation (Coady and Lorson, 2011; Fischer et al., 1997; Meister et al., 2002; Paushkin et al., 2002; Pellizzoni et al., 1999, 2002). Most cases of the disease spinal muscular atrophy are caused by mutations in SMN (Gitlin et al., 2010; Pearn, 1980). Regarding telomerase biogenesis, SMN interacts with hTERT (Bachand et al., 2002), Gar1 (Pellizzoni et al., 2001) and WRAP53 (Mahmoudi et al., 2010). Additionally, previous results have shown that SMN can directly interact with RNA (Bertrandy et al., 1999; Lorson and Androphy, 1998). The functional consequence of these associations is unclear, but strongly suggests that SMN takes part in some aspect of telomerase formation. Another interactor of SMN is coilin, the CB marker protein (Hebert et al., 2002, 2001). Coilin has been shown to interact with WRAP53 (Enwerem et al., 2014; Mahmoudi et al., 2010), and numerous small non-coding RNAs (Machyna et al., 2014). Our work has shown that coilin strongly associates with a subset of box C/D scaRNAs (scaRNA2 and scaRNA9) as well as hTR (Enwerem et al., 2014). We have also found that coilin has RNA processing activity (Broome and Hebert, 2012) that shows specificity towards the 3′-end of pre-processed hTR (Broome et al., 2013; Broome and Hebert, 2012, 2013). The associations of coilin with telomerase components indicate that this protein contributes in some way to telomerase biogenesis.

Despite the fact that CBs have been proposed to facilitate telomerase holoenzyme formation and delivery of telomerase to telomeres (Stern et al., 2012; Venteicher et al., 2009), knockout of coilin (and resultant abolishment of CBs) in HeLa cells has been shown to have no impact on the assembly of telomerase or its trafficking (Chen et al., 2015). It is possible that the KO protocol selects for cells that have adapted or accommodated for the loss of coilin and CBs considering that cells depleted for coilin using RNAi proliferate more slowly than control treated cells (Lemm et al., 2006), but the coilin-KO cells proliferate at the same rate as cells expressing coilin (Chen et al., 2015). Alternatively, the coilin-KO results may indicate that other telomerase assembly factors normally enriched within the CB can still function efficiently in the nucleoplasm when this subnuclear domain is absent. Another possibility is that factors enriched within the CB both positively and negatively regulate telomerase biogenesis. To explore this hypothesis, and refine our understanding of SMN's and coilin's role in telomerase formation, we examined if these proteins could be found in the telomerase complex by monitoring their association with telomerase core components. We also investigated if SMA patient mutations alter the interaction of SMN with the telomerase complex, and test the effect of SMN, coilin or WRAP53 knockdown on telomerase formation. Collectively, the results of these studies demonstrate that SMN and coilin are present in the dyskerin-hTR complex, and, unlike WRAP53, negatively regulate the association of hTR with dyskerin.

## RESULTS

### Coilin interaction with dyskerin is mediated by hTR

**Fig. 1.**
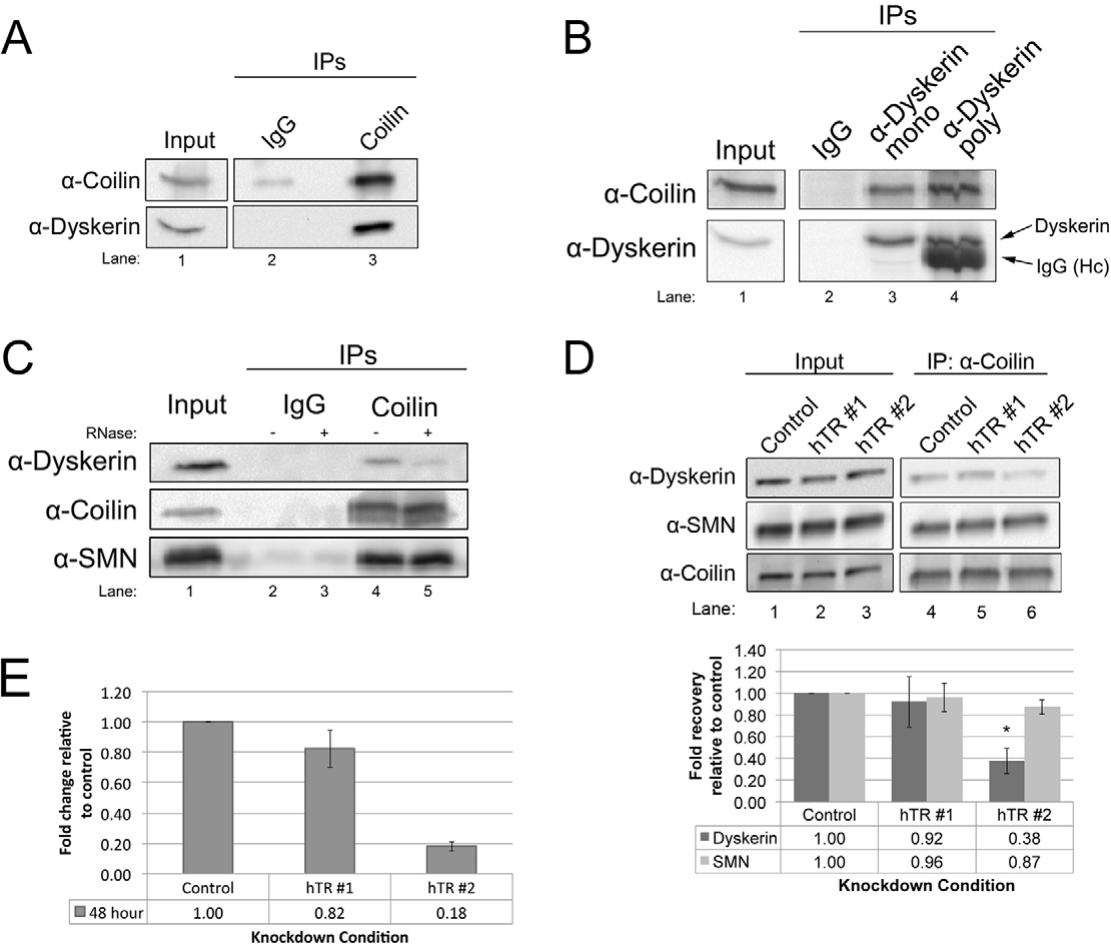
**Coilin interaction with the telomerase complex protein dyskerin is mediated by hTR.** (A) Lysate was immunoprecipitated (IPed) with either control (rabbit IgG) or coilin polyclonal antibodies, followed by running the samples on SDS-PAGE and western transfer. The probing antibodies are indicated. (B) Lysate was IPed with control (mouse IgG), dyskerin monoclonal or dyskerin polyclonal antibodies. Probing antibodies are indicated (Hc in lane 4=IgG heavy chain). (C) Lysate was IPed with control (rabbit IgG) or coilin polyclonal antibodies. Antibody-bead complexes were untreated (−) or treated (+) with RNase. Probing antibodies are indicated. (D) Cells were transfected with two different hTR siRNAs, followed by IP with anti-coilin antibodies. Probing antibodies are indicated. Quantification of dykserin and SMN recovery relative to that obtained with control siRNA for this and other experimental replicates is shown in the lower panel. For all IPs, inputs represent 1.5% of the cell lysate used in the IP reactions. (E) HeLa cells were treated with control or hTR siRNA (hTR #1 or hTR #2) for 48 h. Total RNA was collected and the amount of hTR was analyzed by qRT-PCR, relative to GAPDH message. The level of hTR in cells treated with hTR siRNA was compared to that found in cells with control siRNA for each time point (*n*=3). *P<0.05 compared to control knockdown. For all histograms, error bars denote standard error about the mean.

We have previously shown that coilin can associate with hTR and process this RNA, with specificity towards the 3′-end of the pre-processed transcript (Broome et al., 2013; Broome and Hebert, 2013; Enwerem et al., 2014). To evaluate if coilin also associates with known protein components of telomerase, we conducted a series of co-immunoprecipitation (co-IP) experiments. These experiments also evaluated if SMN interacts with other components of the telomerase complex in addition to the previously reported hTERT (Bachand et al., 2002) and Gar1 (Pellizzoni et al., 2001). In our first line of experimentation, we showed that endogenous coilin can form a complex with the telomerase and box H/ACA scaRNP core protein dyskerin (Fig. 1). This association was observed using either coilin (Fig. 1A) or dyskerin (Fig. 1B) antibodies to IP. Negative control reactions used control IgG, and showed no significant recovery of the indicated proteins. Since we have published that coilin strongly interacts with hTR but not other box H/ACA scaRNAs to the same extent (Enwerem et al., 2014), we next examined if RNA mediates the association of coilin with dyskerin. RNase treatment significantly reduced the amount of dyskerin co-immunoprecipitated with coilin by 62% compared to that obtained in reactions lacking RNase (Fig. 1C, compare the dyskerin signal in lane 4 to that in lane 5; *n*=3, *P*<0.005). These findings demonstrate that RNA mediates, in part, the association of coilin with dyskerin. Again, negative control reactions (lanes 2 and 3) contained control IgG. We next examined if the reduction of hTR would decrease coilin association with dyskerin. As shown in Fig. 1D, the amount of dyskerin associated with coilin does in fact significantly decrease by 60% (*n*=6, *P*<0.05) when cells are treated with siRNA targeting hTR (compare amount of dyskerin present in lane 6 vs to that found in lane 4, histogram). We note that at the time point of this experiment (48 h knockdown), the level of hTR is reduced 20% for the hTR#1 siRNA but hTR is reduced 80% in hTR#2 siRNA-treated cells (Fig. 1E). Together with our RNA sequencing data showing an association between coilin and hTR (Enwerem et al., 2014), the interaction data shown here strongly argue that coilin forms a complex with dyskerin associated with telomerase RNA. However, coilin complex formation with box H/ACA scaRNP-associated dyskerin may account for the some of the dyskerin signal present upon hTR knockdown. Reduction of RNA or hTR did not affect the amount of SMN associated with coilin (Fig. 1C,D).

**Fig. 2.**
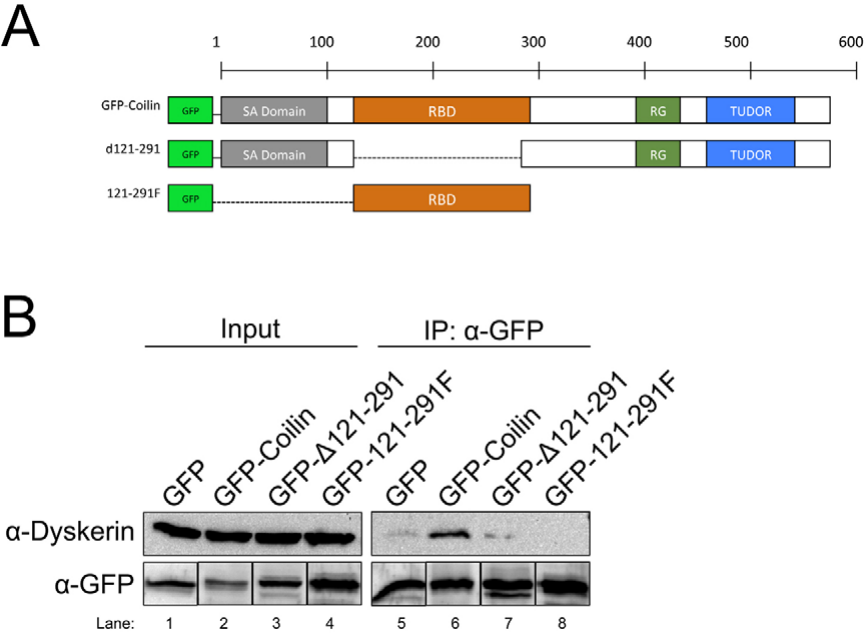
**Mapping of the interaction domain on coilin for association with the dyskerin complex.** (A) Schematic representation of coilin showing the locations of the self-association domain (SA Domain), RNA binding domain (RBD), RG-Box, and TUDOR-like domain (TUDOR) of the various GFP-tagged coilin constructs. (B) Cells were transfected with empty GFP vector or GFP-tagged coilin constructs, followed by IP with antibodies to GFP. Probing antibodies are indicated. For the GFP signals, the same membrane probed with anti-dyskerin was also probed with polyclonal anti-GFP antibodies. The bands for each GFP-tagged protein were then grouped together (lower panel). The experiment was repeated four times, and a representative result is shown. For all IPs, inputs represent 1.5% of cell lysate used in the IP reaction.

We next set out to delineate the region of coilin that is necessary for incorporation into the telomerase complex. For these studies, we utilized coilin fragments fused at the N-terminus to GFP (Fig. 2A). Human coilin is 576 amino acids in length and contains a self-association domain (Hebert and Matera, 2000), an RNA binding/processing domain (Broome and Hebert, 2013), an RG box (Hebert et al., 2001) and a Tudor-like domain (Shanbhag et al., 2010). Cells were transfected with empty GFP vector, plasmids encoding GFP-tagged full-length coilin or fragments thereof. After 24 h, lysates from these cells were subjected to IP with anti-GFP antibodies. As shown in Fig. 2B, GFP-coilin recovers the most amount of dyskerin relative to that recovered by GFP alone. Deletion of the RNA binding/processing domain (RBD, Δ121-291) greatly decreases the amount of dyskerin recovered, but this region alone (GFP-121-291F) is not necessary and sufficient for dyskerin association. These findings demonstrate that coilin association with dyskerin, and by extension, telomerase, requires the RBD of coilin, but amino acids upstream and downstream of the RBD region must facilitate this interaction.

### Novel associations of SMN with components of telomerase

**Fig. 3.**
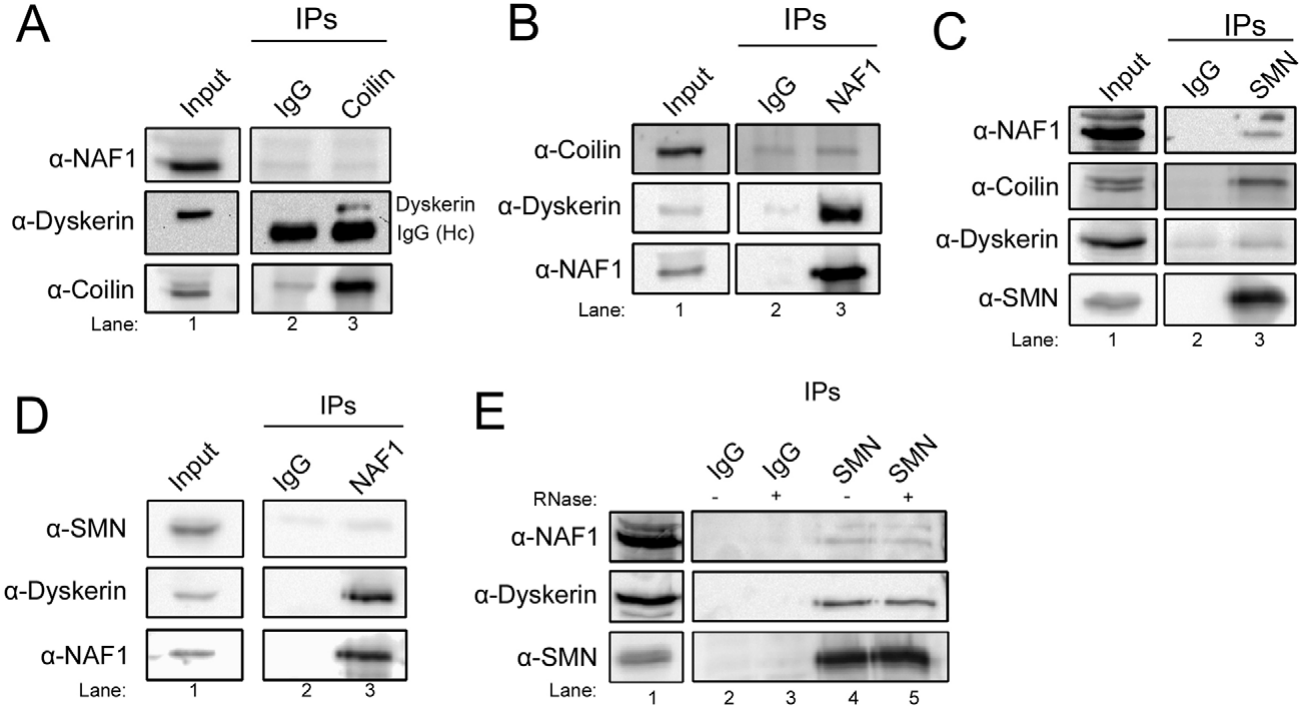
**Differential interaction of coilin and SMN with telomerase complex proteins.** (A) Lysate was IPed with either control (rabbit IgG) or coilin polyclonal antibodies. Probing antibodies are indicated. (B) IP of lysate with either control (rabbit IgG) or NAF1 polyclonal antibodies. Probing antibodies are indicated. (C) Lysate was IPed with either control (mouse IgG) or SMN monoclonal antibodies. Probing antibodies are indicated. (D) The reciprocal experiment of C was performed by IP of lysate with either control (rabbit IgG) or NAF1 polyclonal antibodies. Probing antibodies are indicated. (E) Lysate was IPed with either control (mouse IgG) or SMN monoclonal antibodies. The antibody-bead complexes were untreated (−) or treated (+) with RNase. Probing antibodies are indicated.

To further investigate coilin and telomerase interaction, we examined if coilin associates with the telomerase assembly factor NAF1. No significant recovery of NAF1 or coilin above background (using control IgG) was observed when IPs were conducted with anti-coilin or anti-NAF1 antibodies (Fig. 3A,B), suggesting that coilin is not strongly associated with the NAF1 complex. However, both IP conditions were sufficient to recover dyskerin (middle panels), arguing that coilin forms a complex with dyskerin that lacks NAF1. In contrast, SMN is enriched in the NAF1 complex as shown by IP with anti-SMN (Fig. 3C) or anti-NAF1 (Fig. 3D) antibodies. Negative control reactions using control IgG recover less of the indicated proteins compared to reactions with the appropriate antibody. SMN and coilin have been shown to form a complex previously (Hebert et al., 2001), and Fig. 1C,D and Fig. 3C confirm this interaction. The lack of coilin in NAF1 complexes supports the idea that SMN has many separate complexes, one of which contains NAF1 and lacks coilin and another that contains coilin and lacks NAF1. We also show that the interaction between SMN and NAF1 is not contingent upon RNA (Fig. 3E). Thus, SMN and coilin may both take part in some aspect of telomerase biogenesis given that dyskerin and NAF1 are important factors required to generate functional telomerase. SMN and coilin may contribute to the formation of this RNP by distinct mechanisms given that they associate with different components of the telomerase complex. However, other roles for dyskerin and NAF1 in H/ACA RNP formation may also be affected by interactions with SMN and coilin.

**Fig. 4.**
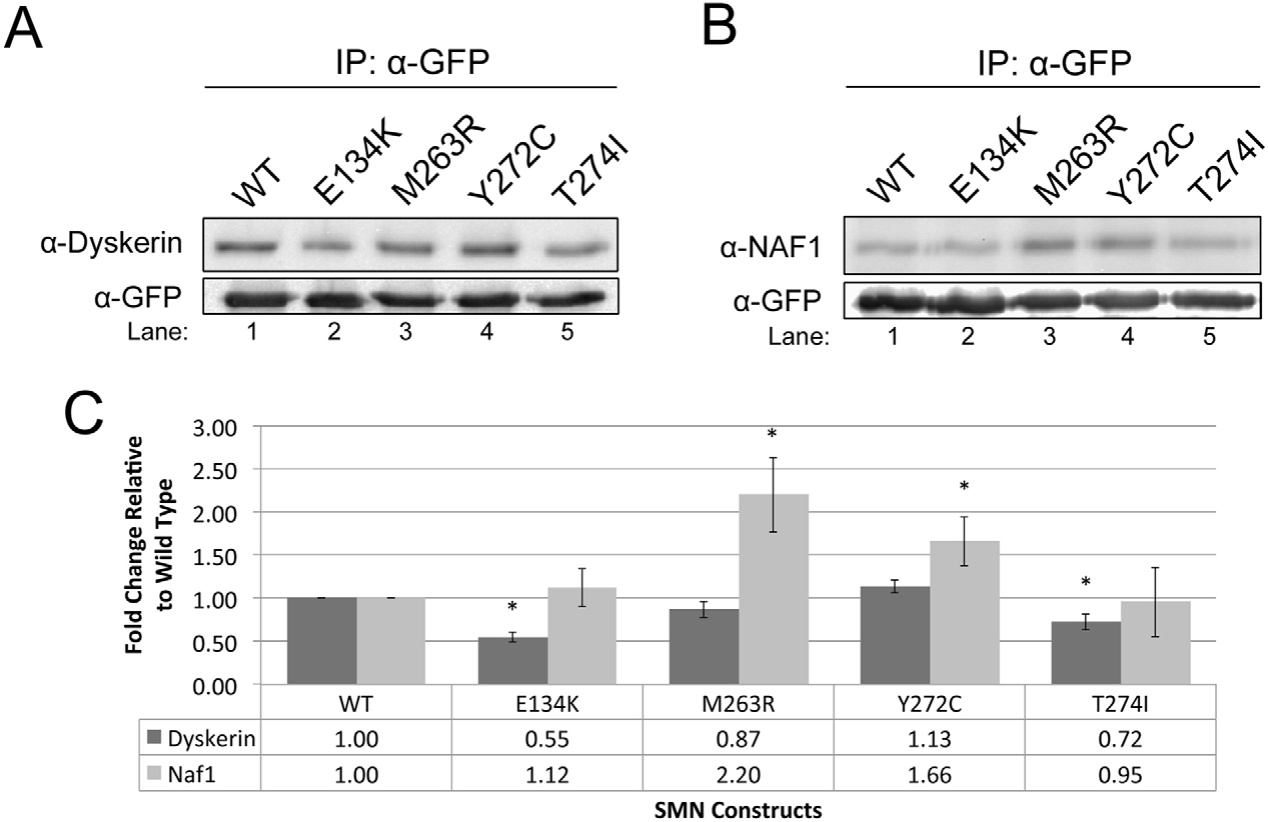
**Clinically defined SMN mutants show alterations in the association with telomerase complex proteins.** (A-B) Cells were transfected with wild-type (WT) or clinically defined SMN mutations, fused to GFP. IPs were performed using anti-GFP antibody. The resulting immunocomplexes were then probed for either dyskerin (A) or NAF1 (B). (C) Graphical representation of the data shown in (A) and (B) (*n*=8), normalized to the recovery of dyskerin and NAF1 by WT SMN. *P<0.05 compared to WT SMN, which serves as a control. Error bars represent standard error about the mean. For all IPs, inputs represent 1.5% the amount of the lysate used in the IP reaction. Note that GFP vector alone does not recover significant amounts of dyskerin (Fig. 2B, lane 5).

Since we observed, for the first time, that SMN associates with dyskerin and NAF1, we next examined if SMN mutations found in patients with SMA are altered in their ability to interact with these telomerase associated proteins. Four clinically defined SMN mutants were tested (E134K, M263R, Y272C and T274I) (Burghes and Beattie, 2009; Praveen et al., 2014). GFP-tagged wild-type (WT) or mutant SMN plasmids were transfected into HeLa cells, followed 24 h later by lysate generation and IP with anti-GFP antibodies. The amount of dyskerin and NAF1 in the SMN complexes was then examined by western blotting. The background level of dyskerin recovered by the GFP vector alone is shown in Fig. 2B, lane 5. As shown in Fig. 4A and B and quantified in Fig. 4C, the SMN mutants differentially associate with dyskerin (Fig. 4A) and NAF1 (Fig. 4B) relative to the amount of these proteins recovered by WT SMN. Specifically, the E134K mutation decreases the amount of dyskerin in the SMN complex by approximately 50%, relative to WT SMN (Fig. 4A, compare the dyskerin signal in lane 2 to that in lane 1, data quantified in C). In contrast, the M263R and Y272C mutations result in an increase in the amount of NAF1 recovered, but do not differentially affect association with dyskerin. Like the E134K mutation, dyskerin association is decreased in the T274I complex but NAF1 levels are unaffected. These findings indicate that, while all SMN mutants disrupt the normal levels of dyskerin or NAF1 associated with SMN, the mutants can be grouped as dyskerin interaction reducing (E134K/T274I) or NAF1 interaction increasing (M263R/Y272C).

### Coilin reduction alters the composition of the dyskerin complex

**Fig. 5.**
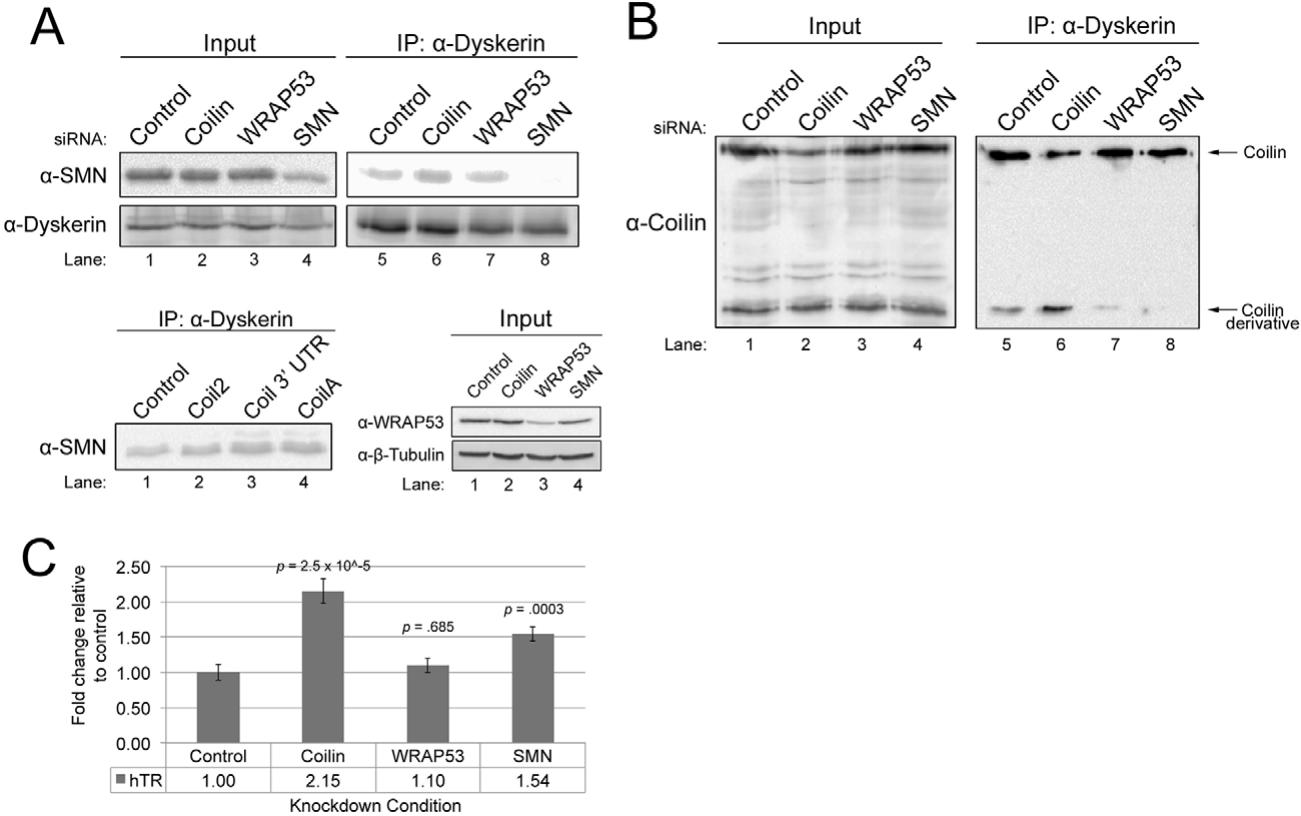
**Reduction of Cajal body proteins alters the composition of the dyskerin complex.** (A, upper panel) siRNA-treated lysate was IPed with anti-dyskerin. A portion of the IPs were subjected to SDS-PAGE, western blotting, and probing with anti-SMN or anti-dyskerin antibodies, while another portion of the IPs was used to isolate RNA for the experiment shown in C. (Lower panel) Three different siRNAs (Coil2, Coilin 3′ UTR or CoilA) were used to knockdown coilin in HeLa cells. Cells were also transfected with control siRNA. 48 h later, lysates were generated and IPed using a dyskerin monoclonal antibody. The IPs were then subjected to SDS-PAGE and western blotting. The membrane was then probed with a SMN antibody. Also shown is the knockdown of WRAP53 we achieve using WRAP53 siRNA compared to control, coilin or SMN siRNA. Western blot was probed with anti-WRAP53 then anti-tubulin antibodies. (B) siRNA-transfected cell lysate was IPed with anti-dyskerin antibody. The IPs were subjected to SDS-PAGE, western blotting and probing with anti-coilin antibodies. The location of full-length coilin and the 28 kDa coilin derivative is indicated. For all IPs, inputs represent 1.5% the amount of the lysate used in the IP reaction. (C) Coilin and SMN negatively regulate hTR association with dyskerin. siRNA-transfected lysate was IPed with dyskerin polyclonal antibody, followed by RNA isolation and qRT-PCR to determine the level of hTR, normalized to that obtained with control siRNA (*P*-values are shown, *n*=4 experimental sets). Error bars represent standard error about the mean.

Because SMN, WRAP53 and coilin have all been shown to associate with each other (Enwerem et al., 2014; Hebert et al., 2001; Mahmoudi et al., 2010), and interact with telomerase proteins (Bachand et al., 2002; Tycowski et al., 2009; Venteicher et al., 2009) (Figs 1–4), we next tested the effect of the reduction of one of these proteins on the association of the other proteins with dyskerin. For these studies, cells were treated with control, coilin, WRAP53 or SMN siRNAs for 48 h, followed by lysate generation and IP with anti-dyskerin antibodies. The siRNAs used here reduce protein levels by at least 80% (Fig. 5A, upper panel, lane 4 and lower right panel, lane 3; Fig. 5B, lane 2) (Enwerem et al., 2014). Interestingly, coilin reduction results in a significant increase (1.5-fold, *n*=5, *P*<0.005) in the amount of SMN recovered in the dyskerin complex (Fig. 5A). This increase of SMN in the dyskerin complex upon coilin reduction was also observed when using two additional coilin siRNAs (Fig. 5A, lower left panel). In contrast, the reduction of WRAP53 did not alter the amount of SMN associated with dyskerin (Fig. 5A), nor did this treatment change the amount of coilin recovered by dyskerin IP (Fig. 5B). Furthermore, SMN knockdown (KD) did not alter the amount of coilin associated with dyskerin (Fig. 5B).

Curiously, we observed an interesting finding when we examined the amount of coilin co-IPed with dyskerin in coilin KD cells (Fig. 5B). We have previously reported that coilin antibodies can recognize a smaller 28-kDa species (Velma et al., 2012). It is possible that this smaller protein recognized by coilin antibodies is a fragment generated from full-length coilin by calpain (Velma et al., 2012). However, it is also possible that this product, denoted as the coilin derivative, is derived from a coilin pseudogene, *COILP1*, which has the potential to encode a 203 aa protein. Current studies are underway to explore this possibility. Coilin siRNAs can reduce the level of the coilin derivative, but require 72 h treatment in order to see clear reduction (Velma et al., 2012). The IP experiments shown here are done with 48 h KD, and, at this time point, the level of full-length coilin is noticeably reduced yet the coilin derivative is not reduced, as expected from our previous studies (Velma et al., 2012) (input lanes, Fig. 5B). When conducting IPs with dyskerin antibodies on lysate from coilin siRNA-treated cells, we observe that the amount of coilin co-IPed is, as expected, reduced. However, we consistently observed that the amount of the coilin derivative recovered in the dyskerin complex was significantly increased 2.88-fold (*P*<0.05, *n*=3) upon coilin siRNA treatment compared to that obtained with control siRNA (Fig. 5B, compare coilin derivative signal in lane 6 to that in lane 5). Interestingly, we also observed that this coilin derivative signal in the dykserin complex was decreased relative to that observed for control siRNA upon WRAP53 or SMN KD (Fig. 5B, compare coilin derivative signal in lanes 7 and 8 to that in lane 5; *n*=3). Based on this data, we conclude that coilin, as well as WRAP53 and SMN, influence the amount of the coilin derivative in the dyskerin complex.

### Dyskerin association with hTR is negatively affected by the Cajal body proteins coilin and SMN

We next examined if KD of coilin, WRAP53 or SMN would impact telomerase assembly by quantifying the amount of hTR present in dyskerin IPs after treatment with siRNAs. For these experiments, cells were treated for 48 h with control, coilin, WRAP53 or SMN siRNAs, followed by lysate generation and IP with anti-dyskerin. After extensive washing of the IP beads, RNA was isolated from the beads and subjected to qRT-PCR to determine to what extent, if any, these knockdowns have on the association of hTR with dyskerin (Fig. 5C). Relative to control KD, reduction of coilin increases the amount of hTR present in the dyskerin complex 2.1-fold. WRAP53 KD did not significantly alter the amount of hTR present. As observed with coilin reduction, SMN KD results in a significant increase in the amount of hTR associated with dyskerin. The amount of dyskerin IPed in these experiments was approximately equal, as shown in Fig. 5A. The data presented in Fig. 5 thus strongly indicate that SMN and coilin negatively regulate hTR incorporation into the dyskerin complex.

### Coilin overexpression correlates with decreased hTR association with dyskerin and reduced telomerase activity

**Fig. 6.**
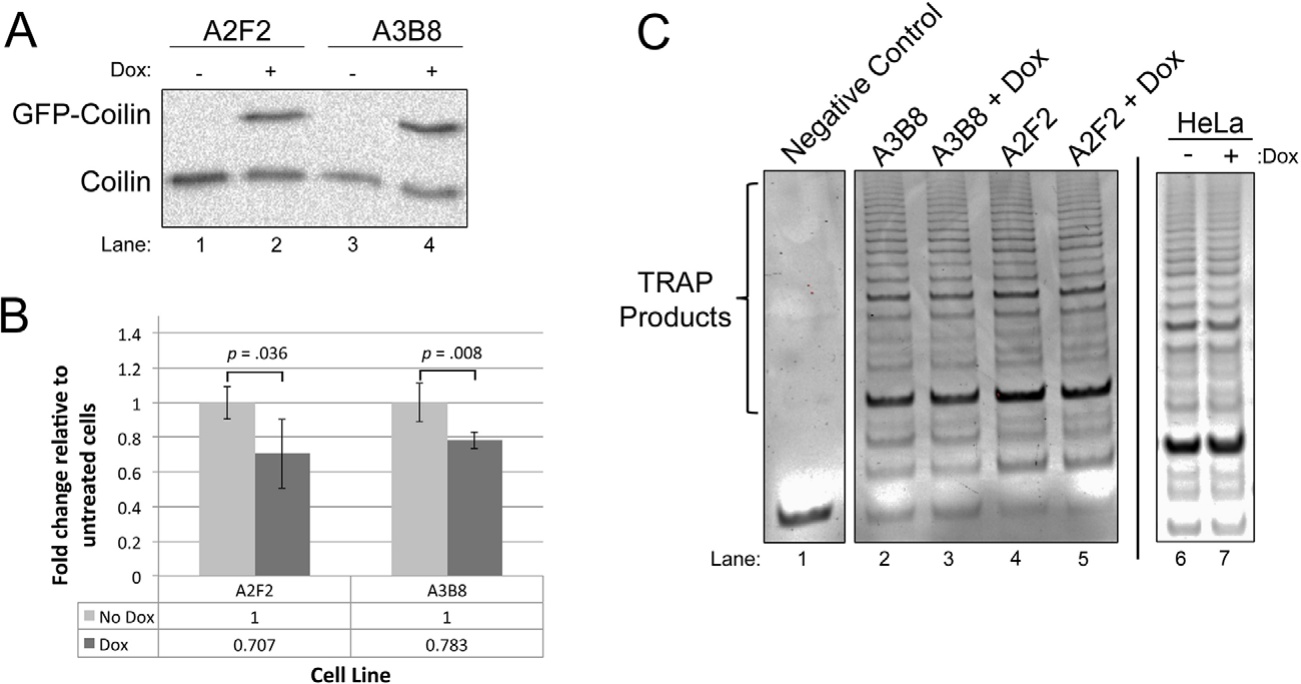
**Coilin overexpression is correlated with decreased association of hTR with dyskerin and reduced telomerase activity.** (A) Verification of Dox induction of GFP-coilin in the A2F2 and A3B8 lines. Cells were untreated or treated for 48 h with Dox, followed by lysate generation, SDS-PAGE, western blotting and probing with anti-coilin antibodies. (B) Lysate from untreated or Dox-treated (48 h) A2F2 or A3B8 cells was IPed with anti-dyskerin, followed by RNA isolation and qRT-PCR to determine the relative amount of hTR. There is a significant decrease in hTR with dox induction compared to uninduced (*P*=0.035 for A2F2 line, *P*=0.008 for A3B8 line, *n*=4). Error bars represent standard error about the mean. (C) Telomerase activity assays were conducted on equal protein amounts from Dox-induced or uninduced lysate from A2F2 and A3B8 lines, and samples were run on an agarose gel and stained with ethidium bromide (lysis buffer served as negative control). Dox induction of GFP-coilin in A2F2 (lanes 4 and 5) resulted in a 20% decrease in the amount of TRAP signal compared to untreated (*n*=5, *P*<0.0005). For the A3B8 line (lanes 2 and 3), Dox induction resulted in a 10% decrease in TRAP signal compared to untreated (*n*=3, *P*<0.05). Note that the treatment of non-inducible HeLa cells with Dox did not alter telomerase activity compared to untreated cells (C, right panel).

Since coilin reduction increased the amount of hTR present in the dyskerin complex, we next tested if coilin overexpression would decrease hTR associated with dyskerin. For this experiment, we utilized two previously characterized doxycycline-inducible coilin cell lines (Carrero et al., 2011). As shown in Fig. 2, GFP-coilin, like endogenous coilin, is capable of associating with dyskerin. The two cell lines (A2F2 and A3B8) were untreated or treated with doxycycline for 48 h (Fig. 6A), followed by lysate generation and IP with anti-dyskerin. RNA was isolated from the IPs and subjected to qRT-PCR to determine the level of hTR in the dyskerin complex. In both cell lines, overexpression of coilin significantly reduced the amount of hTR present in the dyskerin complex (Fig. 6B). Telomerase activity was also examined in these cell lines using the TRAPeze telomerase detection assay, and doxycycline induction of coilin correlated with slightly reduced levels of telomerase activity (Fig. 6C). These data support the hypothesis that coilin can act as a negative regulator of telomerase formation and activity.

## DISCUSSION

### Negative regulatory role of SMN and coilin on telomerase biogenesis

The data presented demonstrate that the Cajal body proteins SMN and coilin associate with components of telomerase. Clinically defined SMN point mutations impact the interaction between SMN and two of these telomerase components, dyskerin and NAF1 (Fig. 4). Specifically, we observe that the E134K and T274I mutations decrease dyskerin interaction but do not affect NAF1 association, while the M263R and Y272C mutations increase NAF1 binding but do not impact dyskerin association. These findings suggest that disrupted telomerase biogenesis may be a component in the constellation of alterations that take place in spinal muscular atrophy. We have also found that reduction of coilin increases the amount of SMN present in the dyskerin complex (Fig. 5A), indicating that coilin may regulate telomerase association with SMN. The data shown in Fig. 5C support the hypothesis that SMN and coilin are negative regulators of telomerase biogenesis considering that the amount of hTR recovered from dyskerin complexes is increased upon coilin or SMN reduction. In support of this hypothesis, coilin overexpression correlated with a reduction in hTR associated with dyskerin and a slight decrease in telomerase activity (Fig. 6). The actual mechanism for this negative regulation by SMN and coilin on the association of dyskerin with hTR, and subsequent formation of telomerase, awaits further study. Given that SMN and coilin both interact with RNA (Bertrandy et al., 1999; Lorson et al., 1998; Broome et al., 2013; Broome and Hebert, 2013; Enwerem et al., 2014), it is possible that these two proteins help coordinate the activity of telomerase biogenesis factors such as NAF1. Our studies increase the list of telomerase components known to interact with SMN. These include GAR1 (Pellizzoni et al., 2001), hTERT (Bachand et al., 2002), WRAP53 (Mahmoudi et al., 2010), dyskerin (this study) and NAF1 (this study). Importantly, SMN interaction with dyskerin, NAF1 (Fig. 3) or hTERT (Bachand et al., 2002) is not mediated by RNA, suggesting that SMN imparts its influence on telomerase formation via protein:protein interactions.

A recent study using a coilin knockout HeLa cell line found that the assembly of telomerase and targeting of the telomerase complex to telomeres was not affected by the absence of coilin and Cajal bodies (Chen et al., 2015). The findings we present here are in support of this previous study in that we suggest that coilin acts as a negative regulator of telomerase assembly. Another study has shown that coilin knockdown resulted in a reduction of hTR foci at the telomeres in a low hTERT expressing cell line, 293T (Stern et al., 2012). This same study also showed that 293T cells overexpressing hTERT and hTR regained proper hTR targeting to the telomeres even with coilin knockdown. In contrast to that observed with coilin reduction, telomerase recruitment to telomeres was negatively impacted by decreases in WRAP53, even in cells with hTERT and hTR overexpression (Stern et al., 2012). These findings demonstrate that telomerase can localize to telomeres by different routes, and these routes are most likely influenced by overall cellular demand that in turn alters hTERT expression levels and impacts subnuclear organization. Proteins enriched in the Cajal body, such as WRAP53, SMN and coilin, do not contribute in the exact same manner to impact telomerase localization to telomeres, and our studies presented here support this hypothesis. Further work will be necessary to characterize the coilin derivative and the role that it has, if any, on telomerase formation. Our observations shown in Fig. 5B clearly indicate that the amount of the coilin derivative in the dykserin complex is influenced by coilin, WRAP53 and SMN levels. Smaller coilin bands detected by coilin antibodies were first reported in 1993 (Andrade et al., 1993), and we have shown that these bands may be generated by processing of full-length coilin by calpain (Velma et al., 2012). Interestingly, a pseudogene for coilin, *COILP1*, was identified in 1994 but not thought to produce a full-length coilin protein (Chan et al., 1994). According to NCBI AceView, *COILP1* has the potential to encode a 203 aa protein. Many of these amino acids correspond to the RNA binding domain of coilin (Fig. 2A), suggesting that this *COILP1* protein product, if it exists, has functional redundancy with coilin. We are currently examining this possibility.

**Fig. 7.**
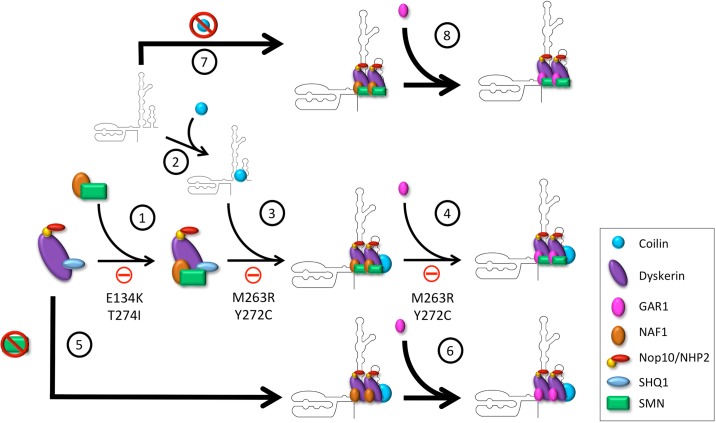
**A model for the negative regulation of SMN and coilin on telomerase biogenesis.** (1) NAF1 and SMN form a complex which is then loaded on to a pre-assembled dyskerin complex containing dyskerin, SHQ1, NHP2, and Nop10. However, the SMN mutations E134K and T274I inhibit this pathway. (2) Coilin binds to nascent hTR near dyskerin's binding site. (3) The dyskerin complex is then loaded onto hTR; however, coilin is not lost in this process and remains in the complex. SMN mutations M263R and Y272C inhibit the association of the dyskerin complex with hTR. (4) GAR1 is exchanged for NAF1 in the dyskerin complex. SMN mutations M263R and Y272C may also inhibit biogenesis at this step by blocking the association of GAR1 with the dyskerin complex. (5) If SMN expression is reduced, the dyskerin complex loads more readily on to hTR (indicated by a larger arrow), and (6) assembly of telomerase proceeds in the absence of SMN. (7) Similarly, if coilin expression is reduced, the dyskerin complex containing SMN will more readily bind hTR (indicated by a larger arrow), (8) and assembly will continue in the absence of coilin. Note: the stoichiometry shown here in regards to SMN and coilin in the telomerase complex is not intended to be definitive. In fact, interactions between SMN and coilin with telomerase components are most likely transient and require sub-stoichiometric amounts.

Overall, the data presented here provide new insights into the differential role that the Cajal body proteins SMN, WRAP53 and coilin may have on telomerase biogenesis. We speculate that SMN and coilin contribute to negatively regulate telomerase biogenesis, possibly at an early step in the formation of this RNP (Fig. 7). Such regulation may ensure that hTR is correctly trafficked and incorporated into a properly assembled premature complex that will become, after several additional steps, the telomerase holoenzyme. In transformed cells that highly express hTERT, such as HeLa, the negative regulation of SMN and coilin on telomerase formation may be dispensable. We further speculate that certain SMN point mutations (E134K, T274I, M263R and Y272C) might disrupt the efficient formation of telomerase given their altered interaction characteristics with dyskerin and NAF1 (Fig. 7). Collectively, our findings provide another layer of knowledge into the biogenesis of telomerase, and the possible role of SMN and coilin in the formation of this RNP. Thus our studies may help to clarify the dyskeratosis congenita and spinal muscular atrophy disease states.

## MATERIALS AND METHODS

### Cell lines and cell culture

HeLa cells were obtained from the American Type Culture Collection (Manassas, VA, USA). A2F2 and A3B8 doxycycline-inducible coilin HeLa cell lines were previously generated (Carrero et al., 2011). Cell lines were cultured in Dulbecco's Modified Eagle's Medium (DMEM) (Invitrogen, Carlsbad, CA, USA) containing 10% fetal calf serum (Atlanta Biologicals, Flowery Branch, GA, USA) and 1% penicillin/streptomycin (ThermoFisher, Waltham, MA) in a 5% CO_2_ incubator at 37°C. A2F2 and A3B8 cell lines were induced with 1 μg doxycycline per ml medium for 48 h.

### RNAi

HeLa cells were transfected with siRNA using Lipofectamine 2000 (Invitrogen, Carlsbad, CA, USA) following the manufacturer's suggested protocol. Unless otherwise noted, siRNA transfections were for 48 h. The siRNAs listed in Table 1 and purchased from Integrated DNA Technologies (Coralville, IA, USA), were used:

**Table 1.**
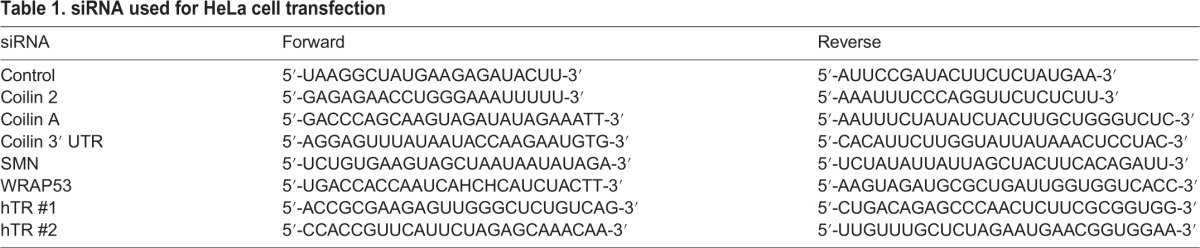
**siRNA used for HeLa cell transfection**

### Plasmids and transfection

HeLa cells were transfected with DNA using FuGENE HD (Promega, Madison, WI, USA) following the manufacturer's suggested protocol. GFP-Coilin (Hebert and Matera, 2000), GFP-Coilin-Δ121-291, and GFP-Coilin-121-291 (Broome and Hebert, 2013) were previously described. To generate SMN point mutations, GFP-SMN (a gift from Greg Matera, University of North Carolina at Chapel Hill, USA), was subjected to PCR amplification using Herculase II Fusion DNA Polymerase (Agilent, Santa Clara, CA, USA) following the manufacturer's suggested protocol with mutagenesis primers. The primers (Integrated DNA Technologies, Coralville, IA, USA) listed in Table 2 were used to generate each point mutation.

**Table 2.**
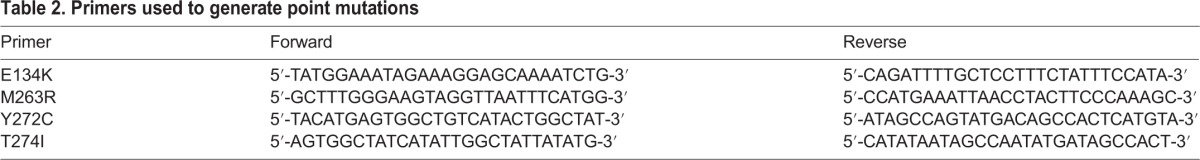
**Primers used to generate point mutations**

### Antibodies

The following antibodies were used in this study: GFP monoclonal (11814460001, Roche, Indianapolis, IN, USA), SMN monoclonal (610646, BD Transduction Laboratories, Franklin Lakes, NJ, USA), Coilin polyclonal (sc-32860, Santa Cruz Biotechnology, Santa Cruz, CA, USA), Dyskerin monoclonal (sc-373956, Santa Cruz Biotechnology), Dyskerin polyclonal (sc-48794, Santa Cruz Biotechnology), NAF1 polyclonal (A303-911A, Bethyl Laboratories, Montgomery, Texas, USA), Normal Mouse IgG (sc-2025, Santa Cruz Biotechnology), and Normal Rabbit IgG (sc-2027, Santa Cruz Biotechnology).

### Immunoprecipitation

HeLa cells were lysed in mRIPA buffer (50 mM Tris-HCl pH 7.6, 150 mM NaCl, 1% NP-40, 0.25% sodium deoxycholate, 1 mM EDTA) containing protease inhibitor cocktail (Roche, Indianapolis, IN, USA) on ice for 10 min. The lysates were sonicated three times in 5-s intervals at 5 W using the Fisher Scientific Sonic Dismembrator Model 100. The lysates were then clarified by centrifugation at 16,000 ***g*** for 15 min at 4°C. 15 μl of the cleared lysate from each sample was saved for input and the remainder was incubated for one hour at 4°C while rocking with 2 μg of antibody. Then, the cleared lysates were incubated for two hours at 4°C while rocking with 40 μl 50% Protein G Sepharose 4 Fast Flow beads (GE Healthcare, Pittsburg, PA, USA). The antibody-bead complexes were then washed three times with 1 ml mRIPA buffer following centrifugation at 4500 ***g***. Beads were resuspended in 2× SDS loading buffer and subjected to SDS-PAGE and western transfer.

### RNase treatment

Where indicated, immunocomplexes were treated with RNase AT1 (AM286, Life Technologies, Grand Island, NY, USA) for 30 min at 37°C in PBS, and were then washed three times with mRIPA buffer. The beads were then resuspended in 2× SDS loading buffer and loaded onto an SDS-PAGE followed by western transfer.

### RNA immunoprecipitation

HeLa cells were lysed in mRIPA buffer (50 mM Tris-HCl pH 7.6, 150 mM NaCl, 1% NP-40, 0.25% sodium deoxycholate, 1 mM EDTA) containing protease inhibitor cocktail (Roche). The lysates were sonicated three times in 5-s intervals at 5 W using the Fisher Scientific Sonic Dismembrator Model 100. The lysates were then cleared by centrifugation at 16,000 ***g*** for 15 min at 4°C. 15 μl of the cleared lysate from each sample was saved for input and the remainder was incubated for one hour at 4°C while rocking with 2 μg of either normal rabbit IgG (sc-2027) or anti-Dyskerin (sc-48794) obtained from Santa Cruz Biotechnology. Then, the cleared lysates were incubated for two hours at 4°C while rocking with 40 μl 50% Protein G Sepharose 4 Fast Flow beads (GE Healthcare). The antibody-bead complexes were then washed three times with 1 ml mRIPA buffer following centrifugation at 4500 ***g***. Approximately 30% of the beads were used to isolate bound RNA for qRT-PCR analysis, and the remaining 70% were used for western blot analysis.

### Western blot

Cell lysate or immunocomplexes were re-suspended in 2× SDS lysis buffer and boiled at 95°C for 5 min. Then, the samples were subjected to SDS-PAGE and transferred to a nitrocellulose membrane. The membrane was then blocked with 5% non-fat milk and TBST (50 mM Tris, 150 mM NaCl, 0.05% Tween 20). Membranes were then immunoblotted with antibodies in 2.5% non-fat milk and TBST. Bands were detected by incubating the membranes with species-specific HRP-conjugated antibodies in 2.5% non-fat milk and TBST for 1 h followed by 5 min incubation with SuperSignal West Pico Chemiluminescent Substrate (#34080, Life Technologies). All membranes were imaged using a ChemiDoc imager with Quantity One software (BioRad, Hercules, CA, USA).

### RNA isolation

RNA was isolated from the antibody-bead complexes using the Machery-Nagel NucleoSpin RNA II kit (Clontech, Mountain View, CA, USA). The RNA isolation was performed by re-suspending the antibody-bead complexes in 350 μl Buffer RA1+3.5 μl β-mercaptoethanol. The bead slurry was then centrifuged for 3 min at 4500 ***g*** after which the supernatant was saved, and the manufacturer's suggested protocol was then followed beginning with ethanol precipitation. RNA isolated from cells was performed per the manufacturer's suggested protocol.

### qRT-PCR

Brilliant II SYBR Green quantitative reverse transcriptase RT-PCR Master Mix kit from Agilent (Santa Clara, CA, USA) was used for the analysis of hTR RNA present in immunoprecipitation experiments. RNA from the RNA immunoprecipitation was used in equal volumes, and qRT-PCR was performed using standard molecular biology techniques. GAPDH message was amplified to serve as a normalizer. Primer sequences to amplify the human telomerase RNA component (hTR) are: Forward, 5′-AAATGTCAGCTGGCCCGTTCG-3′; Reverse, 5′-ACCCGCGGCTGACAGAGCCCAAC-3′; and were obtained from Integrated DNA Technologies (Coralville, IA, USA).

### Telomerase activity assay

Lysate was obtained from uninduced or Dox-treated cells after 48 h. exposure. Protein levels were quantified using the BCA protein assay reagent (Pierce, Rockford, IL, USA), and equal protein amounts were used to determine telomerase activity. Telomerase activity was measured using the telomere repeat amplification protocol with the TRAPeze Telomerase Detection Kit (S27700; Millipore, Billerica, MA, USA) following the manufacturer's suggested protocol.

## References

[BIO018804C1] Andrade L. E. C., Tan E. M. and Chan E. K. L. (1993). Immunocytochemical analysis of the coiled body in the cell cycle and during cell proliferation. *Proc. Natl. Acad. Sci. USA* 90, 1947-1951. 10.1073/pnas.90.5.1947 8446613 PMC45997

[BIO018804C2] Bachand F., Boisvert F.-M., Cote J., Richard S. and Autexier C. (2002). The product of the survival of motor neuron (SMN) gene is a human telomerase-associated protein. *Mol. Biol. Cell* 13, 3192-3202. 10.1091/mbc.E02-04-0216 12221125 PMC124152

[BIO018804C3] Bertrandy S., Burlet P., Clermont O., Huber C., Fondrat C., Thierry-Mieg D., Munnich A. and Lefebvre S. (1999). The RNA-binding properties of SMN: deletion analysis of the zebrafish orthologue defines domains conserved in evolution. *Hum. Mol. Genet.* 8, 775-782. 10.1093/hmg/8.5.775 10196366

[BIO018804C4] Broome H. J. and Hebert M. D. (2012). In vitro RNase and nucleic acid binding activities implicate coilin in U snRNA processing. *PLoS ONE* 7, e36300 10.1371/journal.pone.0036300 22558428 PMC3338655

[BIO018804C5] Broome H. J. and Hebert M. D. (2013). Coilin displays differential affinity for specific RNAs in vivo and is linked to telomerase RNA biogenesis. *J. Mol. Biol.* 425, 713-724. 10.1016/j.jmb.2012.12.014 23274112 PMC3568234

[BIO018804C6] Broome H. J., Carrero Z. I., Douglas H. E. and Hebert M. D. (2013). Phosphorylation regulates coilin activity and RNA association. *Biol. Open* 2, 407-415. 10.1242/bio.20133863 23616925 PMC3625869

[BIO018804C7] Burghes A. H. M. and Beattie C. E. (2009). Spinal muscular atrophy: why do low levels of survival motor neuron protein make motor neurons sick? *Nat. Rev. Neurosci.* 10, 597-609. 10.1038/nrn2670 19584893 PMC2853768

[BIO018804C8] Carrero Z. I., Velma V., Douglas H. E. and Hebert M. D. (2011). Coilin phosphomutants disrupt Cajal body formation, reduce cell proliferation and produce a distinct coilin degradation product. *PLoS ONE* 6, e25743 10.1371/journal.pone.0025743 21991343 PMC3185009

[BIO018804C9] Chan E. K. L., Takano S., Andrade L. E. C., Hamel J. C. and Matera A. G. (1994). Structure, expression and chromosomal localization of human p80-coilin gene. *Nucleic Acids Res.* 22, 4462-4469. 10.1093/nar/22.21.4462 7971277 PMC308480

[BIO018804C10] Chen Y., Deng Z., Jiang S., Hu Q., Liu H., Songyang Z., Ma W., Chen S. and Zhao Y. (2015). Human cells lacking coilin and Cajal bodies are proficient in telomerase assembly, trafficking and telomere maintenance. *Nucleic Acids Res.* 43, 385-395. 10.1093/nar/gku1277 25477378 PMC4288172

[BIO018804C11] Coady T. H. and Lorson C. L. (2011). SMN in spinal muscular atrophy and snRNP biogenesis. *Wiley Interdiscip. Rev. RNA* 2, 546-564. 10.1002/wrna.76 21957043

[BIO018804C12] Egan E. D. and Collins K. (2012). Biogenesis of telomerase ribonucleoproteins. *RNA* 18, 1747-1759. 10.1261/rna.034629.112 22875809 PMC3446700

[BIO018804C13] Enwerem I. I., Velma V., Broome H. J., Kuna M., Begum R. A. and Hebert M. D. (2014). Coilin association with Box C/D scaRNA suggests a direct role for the Cajal body marker protein in scaRNP biogenesis. *Biol. Open* 3, 240-249. 10.1242/bio.20147443 24659245 PMC3988793

[BIO018804C14] Fischer U., Liu Q. and Dreyfuss G. (1997). The SMN-SIP1 complex has an essential role in spliceosomal snRNP biogenesis. *Cell* 90, 1023-1029. 10.1016/S0092-8674(00)80368-2 9323130

[BIO018804C15] Gitlin J. M., Fischbeck K., Crawford T. O., Cwik V., Fleischman A., Gonye K., Heine D., Hobby K., Kaufmann P., Keiles S. et al. (2010). Carrier testing for spinal muscular atrophy. *Genet. Med.* 12, 621-622. 10.1097/GIM.0b013e3181ef6079 20808230 PMC4277882

[BIO018804C16] Hebert M. D. and Matera A. G. (2000). Self-association of coilin reveals a common theme in nuclear body localization. *Mol. Biol. Cell* 11, 4159-4171. 10.1091/mbc.11.12.4159 11102515 PMC15064

[BIO018804C17] Hebert M. D., Szymczyk P. W., Shpargel K. B. and Matera A. G. (2001). Coilin forms the bridge between Cajal bodies and SMN, the spinal muscular atrophy protein. *Genes Dev.* 15, 2720-2729. 10.1101/gad.908401 11641277 PMC312817

[BIO018804C18] Hebert M. D., Shpargel K. B., Ospina J. K., Tucker K. E. and Matera A. G. (2002). Coilin methylation regulates nuclear body formation. *Dev. Cell* 3, 329-337. 10.1016/S1534-5807(02)00222-8 12361597

[BIO018804C19] Lemm I., Girard C., Kuhn A. N., Watkins N. J., Schneider M., Bordonne R. and Luhrmann R. (2006). Ongoing U snRNP biogenesis is required for the integrity of Cajal bodies. *Mol. Biol. Cell* 17, 3221-3231. 10.1091/mbc.E06-03-0247 16687569 PMC1483051

[BIO018804C20] Lorson C. L. and Androphy E. J. (1998). The domain encoded by exon 2 of the survival motor neuron protein mediates nucleic acid binding. *Hum. Mol. Genet.* 7, 1269-1275. 10.1093/hmg/7.8.1269 9668169

[BIO018804C21] Lorson C. L., Strasswimmer J., Yao J.-M., Baleja J. D., Hahnen E., Wirth B., Le T., Burghes A. H. M. and Androphy E. J. (1998). SMN oligomerization defect correlates with spinal muscular atrophy severity. *Nat. Genet.* 19, 63-66. 10.1038/ng0598-63 9590291

[BIO018804C22] Machyna M., Heyn P. and Neugebauer K. M. (2013). Cajal bodies: where form meets function. *Wiley Interdiscip. Rev. RNA* 4, 17-34. 10.1002/wrna.1139 23042601

[BIO018804C23] Machyna M., Kehr S., Straube K., Kappei D., Buchholz F., Butter F., Ule J., Hertel J., Stadler P. F. and Neugebauer K. M. (2014). The coilin interactome identifies hundreds of small noncoding RNAs that traffic through Cajal bodies. *Mol. Cell* 56, 389-399. 10.1016/j.molcel.2014.10.004 25514182

[BIO018804C24] Mahmoudi S., Henriksson S., Weibrecht I., Smith S., Soderberg O., Stromblad S., Wiman K. G. and Farnebo M. (2010). WRAP53 is essential for Cajal body formation and for targeting the survival of motor neuron complex to Cajal bodies. *PLoS Biol.* 8, e1000521 10.1371/journal.pbio.1000521 21072240 PMC2970535

[BIO018804C25] Meister G., Eggert C. and Fischer U. (2002). SMN-mediated assembly of RNPs: a complex story. *Trends Cell Biol.* 12, 472-478. 10.1016/S0962-8924(02)02371-1 12441251

[BIO018804C26] Paushkin S., Gubitz A. K., Massenet S. and Dreyfuss G. (2002). The SMN complex, an assemblyosome of ribonucleoproteins. *Curr. Opin. Cell Biol.* 14, 305-312. 10.1016/S0955-0674(02)00332-0 12067652

[BIO018804C27] Pearn J. (1980). Classification of spinal muscular atrophies. *Lancet* 315, 919-922. 10.1016/S0140-6736(80)90847-8 6103267

[BIO018804C28] Pellizzoni L., Charroux B. and Dreyfuss G. (1999). SMN mutants of spinal muscular atrophy patients are defective in binding to snRNP proteins. *Proc. Natl. Acad. Sci. USA* 96, 11167-11172. 10.1073/pnas.96.20.11167 10500148 PMC18005

[BIO018804C29] Pellizzoni L., Baccon J., Charroux B. and Dreyfuss G. (2001). The survival of motor neurons (SMN) protein interacts with the snoRNP proteins fibrillarin and GAR1. *Curr. Biol.* 11, 1079-1088. 10.1016/S0960-9822(01)00316-5 11509230

[BIO018804C30] Pellizzoni L., Yong J. and Dreyfuss G. (2002). Essential role for the SMN complex in the specificity of snRNP assembly. *Science* 298, 1775-1779. 10.1126/science.1074962 12459587

[BIO018804C31] Praveen K., Wen Y., Gray K. M., Noto J. J., Patlolla A. R., Van Duyne G. D. and Matera A. G. (2014). SMA-causing missense mutations in survival motor neuron (Smn) display a wide range of phenotypes when modeled in Drosophila. *PLoS Genet.* 10, e1004489 10.1371/journal.pgen.1004489 25144193 PMC4140637

[BIO018804C32] Shanbhag R., Kurabi A., Kwan J. J. and Donaldson L. W. (2010). Solution structure of the carboxy-terminal Tudor domain from human Coilin. *FEBS Lett.* 584, 4351-4356. 10.1016/j.febslet.2010.09.034 20875822

[BIO018804C33] Stern J. L., Zyner K. G., Pickett H. A., Cohen S. B. and Bryan T. M. (2012). Telomerase recruitment requires both TCAB1 and Cajal bodies independently. *Mol. Cell. Biol.* 32, 2384-2395. 10.1128/MCB.00379-12 22547674 PMC3434490

[BIO018804C34] Trahan C. and Dragon F. (2009). Dyskeratosis congenita mutations in the H/ACA domain of human telomerase RNA affect its assembly into a pre-RNP. *RNA* 15, 235-243. 10.1261/rna.1354009 19095616 PMC2648702

[BIO018804C35] Tycowski K. T., Shu M.-D., Kukoyi A. and Steitz J. A. (2009). A conserved WD40 protein binds the Cajal body localization signal of scaRNP particles. *Mol. Cell* 34, 47-57. 10.1016/j.molcel.2009.02.020 19285445 PMC2700737

[BIO018804C36] Velma V., Broome H. J. and Hebert M. D. (2012). Regulated specific proteolysis of the Cajal body marker protein coilin. *Chromosoma* 121, 629-642. 10.1007/s00412-012-0387-4 23064547 PMC3519941

[BIO018804C37] Venteicher A. S., Abreu E. B., Meng Z., McCann K. E., Terns R. M., Veenstra T. D., Terns M. P. and Artandi S. E. (2009). A human telomerase holoenzyme protein required for Cajal body localization and telomere synthesis. *Science* 323, 644-648. 10.1126/science.1165357 19179534 PMC2728071

